# Early Diagnosis of Hearing Loss in Patients Under Methadone Maintenance Treatment

**DOI:** 10.3389/fneur.2019.00749

**Published:** 2019-07-16

**Authors:** Arash Bayat, Nader Saki, Golshan Mirmomeni, Ali Yadollahpour

**Affiliations:** ^1^Hearing Research Center, Imam Khomeini Hospital, Ahvaz Jundishapur University of Medical Sciences, Ahvaz, Iran; ^2^Department of Biostatistics and Epidemiology, School of Health, Ahvaz Jundishapur University of Medical Sciences, Ahvaz, Iran; ^3^Department of Medical Physics, School of Medicine, Ahvaz Jundishapur University of Medical Sciences, Ahvaz, Iran

**Keywords:** hearing loss, methadone, pure tone audiometry, extended high frequency audiometry, otoacoustic emission

## Abstract

**Background and Objective:** Methadone maintenance treatment (MMT) as the most effective treatment for opioid addictions could induce both reversible and permanent hearing loss. Therefore, early detection of methadone-induced hearing loss is necessary to prevent irreversible cochlear damage. The present study aims to identify the early onset of hearing loss in patients who underwent MMT and to compare them with the age and gender matched normal hearing peers.

**Methods:** This was an analytic cross-sectional study conducted on patients (*n* = 27 males; age range: 18–53 years old) who received 3 months MMT course (MMT group) and a control group consisting of age and gender matched healthy individuals (*n* = 27 males). Before MMT, all patients underwent conventional audiometry (250–8,000 Hz) and those with normal hearing threshold participated into the study. One month after MMT termination, the patients were assessed for possible hearing loss using conventional pure tone audiometry (PTA), extended high frequency (EHF) audiometry, and distortion product otoacoustic emissions (DPOAEs).

**Results:** Our results demonstrated that the mean EHF thresholds in the MMT patients were significantly greater than the age- and gender-matched healthy controls across all frequencies (*p* < 0.001). However, there was no statistically significant difference in conventional PTA thresholds between both groups (*p* > 0.05). DPOAE amplitudes significantly reduced at higher frequencies (3,000–8,000 Hz) in the MMT group, compared to the healthy control group. In contrast to the conventional PTA audiometry, the EHF and DPOAE assessments identified hearing impairments in 11 (40.74%), and 14 (51.85%) of the MMT patients, respectively. The main mechanisms proposed for methadone induced hearing loss are cochlear ischemia following vasospasm or vasculitis, direct effect of opioids on opioid receptors present in cochlear stria vascularis of inner ear, blood-labyrinth selective transport of opioidproteins and receptors, and genetic polymorphism and mutations.

**Conclusion:** The EHF and DPOAE tests have the potential to detect earlier changes in auditory function than conventional frequency audiometry in the MMT patients.

## Introduction

Methadone has been used for many years in the clinical setting for detoxification treatment of opioid addiction, maintenance treatment of opioid addiction, and as a treatment option for moderate to severe pain (1). Methadone maintenance treatment (MMT) is among the most effective approaches for treatment of opioid addictions which is a maintenance-oriented, rather than abstinence-oriented approach. The common side effects of methadone are well-described and include sedation, constipation, respiratory depression, lightheadedness, dizziness, nausea, and vomiting. Other side effects include dysrhythmias, itching, sweating, and orthostatic hypotension (1–4).

Some studies have reported ototoxicity as a known but rare consequence of opioid medications (3–6). Moreover, it has been shown that opioid consumption could lead to dysfunction at the level of the cochlea or the neural pathways to the auditory cortex, which could either be of sudden or gradual onset (7, 8).

Pure-tone audiometry (PTA) is the most common procedure used to evaluate hearing sensitivity of an individual, which can determine the degree and type of hearing loss. During “conventional” auditory testing, PTA is used to identify hearing threshold in frequencies from 250 to 8,000 Hz. In addition to conventional PTA, extra high frequency (EHF) audiometry technique identifies hearing thresholds within the range of 9,000–18,000 Hz (9). The EHF assessment has been used for early detection of hearing loss attributable to lesions located in the base of the cochlear duct and may indicate alterations even before the characteristic effects appear in the conventional frequency range like noise induced hearing loss and ototoxicity (10).

Otoacoustic emissions (OAEs) are sounds measured in the external ear canal and a reflection of active processes in the cochlea. Initiation and physical characteristics of OAE depend on the physiological status of the outer hair cells (OHCs); thus, OAE testing could serve as an objective measure of ototoxic damage. OAEs can provide valuable information on the integrity of the OHCs within the cochlea and can be utilized for early identification of hearing impairments (11–13). Changes in the functions of OHC alter OAE responses and hearing thresholds. Distortion product OAE (DPOAE) is generated using two stimulating tones, *f*_1_ and *f*_2_ (where *f*_1_ < *f*_2_). The response is initiated in the overlapping region of the basilar membrane's response to the stimuli, somewhat nearer to the *f*_2_ tonotopic place. A second component arises near the basilar membrane place that codes the distortion-product (DP) frequency (2*f*_1_ – *f*_2_). These two sources are combined within the ear canal and the resulting energy is measured as the clinical DPOAEs and may include generator sources basal to the primary tones. The presence of DPOAEs are generally associated with normal hearing and are reduced in individuals with mild to moderate hearing losses up to ~50–60 dB HL. In subjects with hearing thresholds greater than about 60 dB HL, DPOAEs are not generated (14, 15).

Furthermore, studies conducted on the patients who had been treated with ototoxic medications have shown that EHF audiometry and DPOAE measurements can detect evidence of ototoxicity in the inner ear earlier than conventional audiometry, potentially serving as a predictor to ototoxic effects prior to damage occurs in frequencies that are critical for speech and language comprehension (10, 16, 17).

Several studies have reported the impacts of methadone as an ototoxic medication on auditory system but most of these studies were case reports (2–4, 8, 18). The clinical findings have suggested that methadone administration could lead to both reversible and irreversible hearing loss. Prospective monitoring of these ototoxic effects could reduce the hearing impairments. Early detection of hearing loss provides the necessary information to prevent or minimize the hearing loss progression. Moreover, early detection provides an opportunity to implement aural rehabilitation to impede the hearing impairments. To the best of our knowledge, all of the published reports on methadone associated hearing loss were case reports and there is no clinical trials conducted on a sample of patients with a control group. We could find six case reports published on hearing loss after methadone overdose or abuse (2–4, 18). Of them, four cases were reported to show speedy and fully recovery and only two case reports showed persistent hearing loss (4, 8). All of the cases were related to the patients with methadone overdose or abuse. Considering the wide spread administration of MMT for addiction treatments and the recently reported cases of methadone associated hearing loss, investigating the possible causality of methadone to the hearing loss through well-designed clinical trials is necessary.

The current study aims to investigate the diagnostic value of EHF audiometry and DPOAE methods in detecting early onset of hearing loss in the patients addicted to opioid who had underwent MMT. In this regard, the hearing thresholds of the patients were measured using PTA, EHF, and DPOAE techniques and compared against the age and gender matched normal peers.

## Methods

### Study Population

This analytical cross-sectional study was conducted on 27 males (mean age: 35.64 ± 9.25; range: 18–53 years old) with opioid dependence who had been underwent an MMT course in a private clinic. The experiments of the study had lasted from March 2015 to July 2017. To reduce the bias and inter-individual variation we applied a robust inclusion and exclusion criteria. The inclusion criteria were patients who: (i) were at least 18 years of age; (ii) met the DSM-IV-TR or SM-5 criteria for opioid use disorder as assessed by an experienced psychiatrist; (iii) had positive test of opioid dependence in a urine examination before therapy; and (iv) continued to receive MMT for at least three months; (v) were not under any opioid or opioid treatment drugs. During MMT course, all patients were regularly monitored in a private addiction center and prescribed specific dose of methadone. The patients did not use any medication other than methadone for opioid treatment. The control group consisted of 27 healthy males with a similar age distribution (18–53 years of age; mean: 33.95 ± 8.64 years) of the MMT group. All control participants showed normal hearing thresholds (<20 dB HL at 250–8,000 Hz).

All subjects had bilaterally normal middle ear functions before the start of the MMT course. Individuals with a history of chronic exposure to intense noise, acoustic trauma, ototoxic drug consumption, traumatic brain injuries and middle ear disorders were excluded from the study. Furthermore, we excluded those patients with a history of schizophrenia, neurocognitive disorders, neurodevelopmental disorders, major depressive disorders, bipolar disorders, and mental disorders due to medical conditions according to the definitions of DSM-IV-TR or DSM-5.

All the procedures of this study were in complete accordance with the Declaration of Helsinki on ethical principles for medical research involving human subjects (2014) (19). After the enrolment and before the start of the experiments, the researchers clearly explained the procedures, potential benefits and risks of the study to the participants. Written informed consent form was obtained from all participating subjects before the study.

### MMT Course

The MMT course that consisted of daily 30 mg methadone 7 days per week and for 3 consecutive months.

The MMT course was continuous so that patients with interruption in methadone consumption (more than 2 days) were excluded from the study. During the MMT, patients just received methadone and consumption of any other medications was terminated at least 2 months before start of MMT course.

### Hearing Assessment

Before starting MMT all patients underwent conventional audiometry (250–8,000 Hz) and those patients with normal hearing threshold were entered into the study. The audiological assessments (EHF and DPOAE) were conducted 1 month after termination of MMT course.

A clinical two-channel audiometer (Madsen Astera, GN Otometrics, Denmark) coupled with a standard (Telephonic TDH 39, Supra-aural) and extra high-frequency (Sennheiser HDA-200, Circumaural) headphones was used to record hearing thresholds. Conventional audiometry was determined for octave frequencies of 250–8,000 Hz, and EHF audiometry was determined for frequencies of 10,000, 12,000, 14,000, and 16,000 Hz. For EHF assessment, if the participants failed to respond to the maximum intensity produced by the audiometer, the instrument's highest output level was considered as their hearing threshold. All procedures for hearing assessment were carried out in an anechoic chamber according to ISO 8253-1.50 standards.

The DPOAE measurements were performed using a Madsen Capella system (GN Otometrics, Denmark) in an acoustically isolated chamber. Two primary pure tones at frequencies f1 and f2 were utilized, where f1 was set to 65 dB SPL and f2 to 55 dB SPL (f2/f1 ratio = 1.22). DPOAE amplitudes were recorded as a function of f2 frequency at 1,000–6,000 Hz. The acceptance criterion for DOPAE response was set to minimum level of 0 dB SPL and signal to noise ratio of ≥6 dB SPL at each f2 frequency (15).

### Statistical Analysis

Statistical analysis was performed using SPSS version 18 software. Categorical variables were expressed as number and percentage and continuous variables were reported as mean ± standard deviation. Normality of the numerical data was verified using the Kolmogorov–Smirnov (K–S) test. Tests comparing paired data sets were conducted using paired-sample *t*-tests if K-S test indicated that the data were normally distributed. The Wilcoxon signed-rank test for related samples was used if the normality assumption was violated. For all statistical tests, the statistical significance level was set at *p* = 0.05.

## Results

The mean hearing thresholds of the both groups assessed by conventional PTA and EHF are presented in Table 1. The results showed that the mean thresholds at conventional frequencies (250–8,000 Hz) in the MMT group were slightly higher than control group, but these differences were not statistically significant (paired sample *t*-test, *p* > 0.05). In contrary, the mean thresholds for the EHF frequencies in the MMT group significantly increased from 10,000 to 16,000 Hz, compared to the healthy controls (paired sample *t*-test, *p* < 0.001) (Table 1). The mean DPOAE amplitudes for both groups are presented in Table 2. Although the mean DPOAE amplitudes did not differ significantly between the groups at the lower frequencies (≤ 2,000 Hz), the mean amplitudes for frequencies 3,000–8,000 Hz were significantly reduced in the MMT subjects, compared to the controls in both ears.

**Table 1:** Hearing thresholds in normal controls vs. patients underwent methadone maintenance treatment (MMT).

**Group**	**Frequency (Hz)**										
	**250**	**500**	**1,000**	**2,000**	**4,000**	**6,000**	**8,000**	**10,000**	**12,000**	**14,000**	**16,000**
**RIGHT EAR**											
MMT	7.54 (3.92)	11.09 (4.47)	11.45 (4.45)	12.89 (5.78)	13.66 (5.87)	13.30 (7.90)	15.45 (7.25)	26.95 (8.05)	33.90 (12.25)	42.25 (13.46)	54.05 (11.32)
Control	8.39 (3.68)	10.67 (4.95)	12.12 (3.89)	13.45 (5.19)	13.36 (6.34)	14.36 (4.94)	14.66 (6.95)	19.57 (8.58)	22.07 (9.25)	30.90 (10.69)	33.37 (8.90)
*p*-value	NS	NS	NS	NS	NS	NS	NS	<0.001	<0.001	<0.001	<0.001
**LEFT EAR**											
MMT	9.89 (3.17)	10.56 (4.78)	12.49 (4.52)	12.55 (6.23)	13.43 (4.54)	12.35 (7.81)	15.64 (3.12)	27.22 (11.34)	35.54 (8.07)	43.31 (9.25)	49.01 (11.92)
Control	8.54 (3.85)	10.66 (4.54)	11.94 (5.65)	12.28 (4.915)	13.09 (4.71)	11.63 (6.13)	14.31 (6.35)	21.74 (12.45)	25.78 (9.18)	29.45 (10.73)	36.22 (12.14)
*p*-value	NS	NS	NS	NS	NS	NS	NS	<0.001	<0.001	<0.001	<0.001

*All data are expressed as mean (SD) dB HL; NS, Not significant*.

**Table 2:** The DPOAE amplitudes in normal controls vs. patients underwent methadone maintenance treatment (MMT).

**Group**	**Frequency (Hz)**					
	**1,000**	**2,000**	**3,000**	**4,000**	**60,000**	**8,000**
**RIGHT EAR**						
MMT	11.92 (5.62)	10.81 (3.28)	8.44 (5.31)	6.35 (7.08)	3.28 (5.54)	2.79 (3.64)
Control	11.35 (7.80)	11.07 (6.62)	12.75 (5.34)	10.06 (4.76)	8.47 (3.91)	7.38 (4.45)
*p*-value	NS	NS	<0.001	<0.001	<0.001	<0.001
**LEFT EAR**						
MMT	11.38 (5.74)	11.65 (3.90)	7.34 (5.76)	6.79 (4.13)	6.35 (7.08)	1.84 (3.77)
Control	12.06 (5.74)	12.58 (4.54)	10.45 (6.27)	11.23 (5.83)	10.02 (4.76)	6.12 (4.83)
*p*-value	NS	NS	0.031	<0.001	<0.001	<0.001

*All data are expressed as mean (SD) dB SPL; DPOAE, Distortion-Product Otoacoustic Emission; NS, Not significant*.

EHF and DPOAE test, respectively, identified hearing loss in 11 (40.74%), and 14 (51.85%) of the patients who had previously received MMT. Ten (37.04%) patients exhibited abnormal hearing sensitivity in both EHF audiometry and DPOAE ranges in the MMT subjects.

## Discussion

The present study was aimed to identify the early onset of hearing loss in patients who underwent MMT and to compare them with the age matched normal hearing peers. Our findings showed a high prevalence of hearing impairment in patients who received MMT for at least 3 months. The standard method for ototoxicity monitoring is sequential measurement of pure-tone hearing thresholds within the conventional frequency range, 250–8,000 Hz. However, it seems that EHF audiometry and DPOAE technique are more sensitive procedures to detect early damage to auditory system.

All of the 27 enrolled patients who underwent MMT showed normal hearing at frequencies ≤ 8,000 Hz, whereas 11 (40.74%) subjects showed decreased hearing sensitivity at more than one of the four (10,000, 12,000, 14,000, or 16,000 Hz) EHFs. These findings show that methadone firstly damage the basal regions of the cochlea, leading to high frequency bilateral symmetrical sensorineural hearing loss (SNHL). In addition, hearing thresholds analysis in methadone group indicated a significant mild or moderate sloping hearing loss with high variability in EHF thresholds.

DPOAE test is non-invasive, fast and pre-neural procedure that provides frequency- and ear-specific information regarding the integrity of OHCs function of the cochlea. DPOAE assessments are commonly used for newborn hearing screening programs and differential diagnosis of auditory system disorders (14–16). During this study, we observed a significant decline in DPOAE amplitudes in 14 (51.85%) of the patients which was more pronounced in 3,000–8,000 Hz frequency ranges. These findings that the patients in our study who experienced a reduction in DPOAE amplitudes did not demonstrate clinically significant hearing impairment in the 250–8,000 Hz frequency range, implying that DPOAEs can detect subtle cochlear hair cell lesion before hearing loss is appeared in conventional frequency range in patients who underwent methadone therapy. Moreover, our findings indicated that decrements in DPOAE amplitudes occurred at a much faster rate for the higher frequencies than the lower frequencies.

Methadone is a synthetic opioid that is used as an analgesic and as a maintenance anti-addictive medication for patients with opioid dependency. There is a growing body of evidence that abuse of the synthetic opioid can lead to temporary or permanent SNHL (16). So far, six case reports have been published on hearing loss after methadone overdose (2–4, 18). Most of the cases (4 of 6) of hearing loss following methadone abuse have been reportedly shown fully and speedy recovery and only two case reports showed persistent SNHL (4, 8). The findings of these case reports suggested that the reversible or irreversible methadone induced SNHL depends on the duration and time of exposure as well as the health status of drug users (10). Christenson and Marjal (18) reported two patients of sudden SNHL after methadone abuse, though the hearing loss in both cases reversed completely within 24 h. However, none of our patients had history of taking an overdose of prescribed methadone. Most of the previously reported cases of opiate-induced SNHL seem to involve a retrocochlear process. However, there have been few cases of opioids induced SNHL that were improved only after cochlear implants indicating that the chronic opioid induced hearing loss is a cochlear process rather than retrocochlear process (20).

In all cases with speedy recovery the methadone was withheld but in the cases with persistent hearing loss, one case did not stop methadone and used it at prescribed dose (8) and on the other case the data is not available (4). In our study the patients received a fixed dose of methadone (30 mg per day) for three months continuously and then stopped the medication consumption one month before the audiometric assessments. Our findings contradicted the previous case reports in which the patients showed speedy and full resolution following the stop of methadone consumption.

Opioid receptors function in neuronal systems and local networks involved in the initiation of drug action and the subsequent development of adaptations under repeated drug consumption. It has been shown that opioid neuropeptides participate in synaptic processing in inner hair cells of the cochlea (21). The expression of the four opioid receptors (morphine, deferens, ketocyclazocine, and nociceptin), endogenous opioid peptides, and the physiologic response in the cochlea of animal models and in humans suggest that synthetic opioids may influence the homeostasis of the inner ear (16).

Long term consumption of opioids could induce damage in the basal section of the cochlear that could subsequently lead to hearing loss. This damage could increase the hearing threshold especially in higher frequencies and reduce the DPOAE amplitude. This adverse effect of opioid on hearing threshold should be considered in interpretation of our findings. It is possible that the observed higher EHF thresholds and reduced DPOAE amplitudes in the patients with opioid dependency could be due to the basal turn cochlear damage induced by opioid consumptions rather than the effects of MMT.

The pathophysiology of methadone induced hearing loss is not fully understood yet. Different theories have been proposed to explain the effects of opioid drugs on hearing systems (2–4, 8, 16, 22). The most commonly proposed theories are cochlear ischemia, genetic polymorphism and mutations, blood-labyrinth selective transport of proteins and receptors, and direct effect of opioids on opioid receptors.

Cochlear ischemia is mainly occurred following vasospasm or vasculitis of the branch of the spiral modiolar artery. The cochlear structure is very susceptible to hypoxia. Different studies have shown that opioid drugs trigger the production and release of endothelin-1 as an endogenous vasoconstrictor (23). This factor binds to endothelin receptor (ET-A) located on smooth muscle cells of the spiral modiolar artery, and leads to vasospasm and then cochlear ischemia (4).

Genetic polymorphism has been reportedly a possible predisposing factor for opioid-induced hearing impairments particularly aminoglycosides induced hearing impairments. Different mutations and polymorphisms such as allelic variants in the liver metabolic enzymes and mutations induced by mu-opioid receptor (MOR), which are occurred in response to opioid drugs may contribute to different levels of hearing impairments (2).

Blood-labyrinth barrier plays pivotal role in regulating and establishing homeostasis of inner ear fluid mainly through selectively active transport of molecules based on the molecular weight. One hypothesis is based on the inter-person variation in the blood-labyrinth transport channels and differences in the molecular weight of opioid drugs so that in some opioid users opioid drugs are more likely to cross the barrier and induce hearing loss (4, 8).

Other hypothesis on the mechanism of action of opioids in inducing hearing loss is the effect of opioids on specific opioid receptors present in the inner ear. The main opioid receptors present in the central and peripheral nervous systems are mu (MOR), delta (DOR), and kappa (KOR) opioid receptor. The majority of opioids are MOR-agonist. Opioid receptors are present in different structures of the inner ear including inner and OHCs, spiral ganglion, supporting cells of the organ of Corti, and nerve fibers. The activation of MOR can inhibit calcium which in turn inhibits the basal adenylate cyclase activity. Endogenous opioid peptides such as endorphin and enkephalin have likely important role in auditory neuromodulation. These inhibitory effects are more pronounced in cochlear stria vascularis of inner ear, a structure which is rich in blood vessels, and could negatively affect mechanoelectrical transduction of the signal within the cochlea (16, 24, 25). It has been hypothesized that exogenous opioids could stimulate MOR which subsequently impairs endogenous auditory neuromodulation compounds that lead to hearing loss (26, 27). To understand the exact mechanisms of methadone induced hearing loss, further studies should be conducted.

Investigating the risk factors of methadone induced hearing loss, we should consider the mutual relationship between the substance abuse and prevalence of hearing loss. Several studies have suggested that individuals with hearing loss have higher susceptibility to substance abuse. McKee et al. compared the prevalence of substance use disorders among adults with and without self-reported hearing loss among a nationally representative sample (*n* = 86,186) of adults in the US. They reported that hearing loss was independently associated with an increased likelihood of substance abuse disorder. Moreover, hearing loss was independently associated with substance use disorders among age group of ≤ 49 years. Interestingly, these associations are particularly pronounced for prescription opioid use disorders in the group aged 18–34 years (28). However, in our study the subjects showed normal hearing threshold before the start of the MMT; thus, the observed hearing loss could be attributed to the 3 months MMT course. This should be noted that in our study we did not use EHF nor DPOAE for pre-MMT assessments of hearing threshold but we used the conventional PTA for hearing assessments. This limitation could decrease the certainty of the causal link between the MMT and the observed hearing loss.

In conclusion, our findings showed that EHF audiometry and DPOAE techniques have greater diagnostic values than the conventional PTA to detect early changes in auditory functions in patients undergoing MMT. However, opioid addictions could induce damage in the basal section of the cochlear which should be considered in interpretation of our findings. In this regard, it is possible that the observed higher EHF thresholds and reduced DPOAE amplitudes in the patients with opioid dependency may be due to the basal turn cochlear damage induced by opioid consumptions rather than the effects of MMT. Therefore, the effect of methadone treatment cannot be conclusively determined and further case control studies with large sample size with pre- and post-MMT assessments are necessary to shed more light on the effects of methadone on hearing functions.

## Data Availability

The datasets generated for this study are available on request to the corresponding author.

## Ethics Statement

All procedures of this study were approved by Ethics committee of Ahvaz Jundishapur University of Medical Sciences, Ahvaz, Iran.

## Author Contributions

AB, NS, GM, and AY contributed conception and design of the study. AB and NS collected the data and organized the database. GM and AY performed the statistical analysis. AB wrote the first draft of the manuscript. NS, GM, and AY wrote sections of the manuscript. All authors contributed to manuscript revision, read and approved the submitted version.

### Conflict of Interest Statement

The authors declare that the research was conducted in the absence of any commercial or financial relationships that could be construed as a potential conflict of interest.
